# Peyer's Patches Are Precocious to the Appendix in Human Development

**DOI:** 10.1155/2001/71685

**Published:** 2001

**Authors:** S. A. Bhide, K. V. Wadekar, S. A. Koushik

**Affiliations:** 1 Department of Zoology R. T. Marg Civil Lines Nagpur 440 001 India; 2 Department of Zoology Vidarbha Mahavidyalaya Amravati India

**Keywords:** appendix, fetal, human, immunocytochemical, patches, Peyer's

## Abstract

PP are first visible at ∼15.5 wk gestation after which there is a rapid spurt in the development and maturation of lymphoid follicles so that at any given point of time new foci of PP development are continuously formed at a rapid rate. Addition of rows of follicles results in the formation of a PP. Immature PP of younger fetuses have a spongy structure in contrast with the compact lymphoid follicles of mature PP of older fetuses. Immunocytochemical studies reveal that there is a subtle gradation in the expression of lymphocyte surface markers with increasing fetal age. Expression of antigenic markers occurs in an ordered sequence viz. HLA – DR, CD19 (B cell population), CD9 (pre-B cells), CD3 T lymphocytes, CD4 helper / inducer lymphocytes, the CD8 suppressor / cytotoxic cells and lastly, the CD57 Natural Killer cells. The antigens are expressed first on lymphocytes of PP and thereafter in those of the appendix. Our findings clearly demonstrate that the ∼5 wk fetal period from 17.5 wk to 22 wk represents a major growth phase in the development of surface markers of lymphocytes in the mucosal immune system of the gut.


*Developmental Immunology*, 2001, Vol. 8(2), pp. 159-166
Reprints available directly from the publisher
Photocopying permitted by license only

© 2001 OPA (Overseas Publishers Association)
N.V. Published by license under
the Harwood Academic Publishers imprint,
part of The Gordon and Breach Publishing Group,
a member of the Taylor and Francis Group

### Peyer's Patches Are Precocious to the Appendix in Human Development

S.A. BHIDE[a]*, K.V. WADEKAR[b] and S.A. KOUSHIK[c]

*[a]Department of Zoology and [b]Department of Zoology, R. T. Marg, Civil Lines, Nagpur, 440 001, India and [c]Department of Zoology, Vidarbha Mahavidyalaya, Amravati, India*

PP are first visible at ~15.5 wk gestation after which there is a rapid spurt in the development and maturation of lymphoid follicles so that at any given point of time new foci of PP development are continuously formed at a rapid rate. Addition of rows of follicles results in the formation of a PP. Immature PP of younger fetuses have a spongy structure in contrast with the compact lymphoid follicles of mature PP of older fetuses. Immunocytochemical studies reveal that there is a subtle gradation in the expression of lymphocyte surface markers with increasing fetal age. Expression of antigenic markers occurs in an ordered sequence viz. HLA – DR, CD19 (B cell population), CD9 (pre-B cells), CD3 T lymphocytes, CD4 helper / inducer lymphocytes, the CD8 suppressor / cytotoxic cells and lastly, the CD57 Natural Killer cells. The antigens are expressed first on lymphocytes of PP and thereafter in those of the appendix. Our findings clearly demonstrate that the ~5 wk fetal period from 17.5 wk to 22 wk represents a major growth phase in the development of surface markers of lymphocytes in the mucosal immune system of the gut.

*Keywords:* appendix, fetal, human, immunocytochemical, patches, Peyer's

### INTRODUCTION

The gastrointestinal tract is the largest lymphoid organ of the body and is one of the major sites of immunological challenges. Despite this fact, several previous studies have sought to elucidate the role and ontogeny of the mucosal immune system in humans on the basis of studies restricted to the terminal PP only, and to fetuses less than 20 weeks gestation (Spencer *et al*, 1986, Farstad, et al, 1993, 1994, 1995. Halstensen *et al*, 1989, MacDonald *et al*, 1987, Yamamoto, 1988, Bjerke *et al*, 1988, 1993). Such studies, therefore, do not give a true picture of the ontogeny of the mucosal immune system.

To resolve this omission in developmental immunology, the intestine of human fetuses was examined by using an innovative technique in order to ascertain the manner of development of PP hitherto not reported. The gut was also studied to determine the stage wise expression of CD antigens on lymphocytes viz. CD3 – T lymphocytes, CD4 T- helper / inducer cells, CD8 suppresser / cytotoxic T cells, CD19 B and, CD9 pre-B lymphocytes and CD57 Natural Killer cells, simultaneously in the PP as well as the

* Corresponding Author : Dr. (MRS.) S. A. B H I D E Address : Reader, Department of Zoology, R. T. Marg, Civil Lines, Nagpur, 440001, India. Telephone No : 91–712–228090 FAX NO: 91–712–525581 EMAIL : bhidesa@nagpur.dot.net.in appendix of human fetuses. Some aspects of histology and developmental morphology of Peyer's patches not reported so far have also been examined.

### RESULTS

### Formation of Peyer's patches

This is the first detailed record of the stage wise development of PP from the time they first appear at ~15 wk (+ / – 7 days)gestation (Fig. 1a) through ~38 wk (Fig. 1b) gestation. Since very closely graded stages were examined, the most striking feature noticed was the rapidity with which PP develop. A PP begins as a cluster of cells that form a lymphoid follicle. Lymphoid follicles are continuously added until the PP comprises of 3 to 5 rows of lymph nodules, rectangular, oblong or circular in shape (Fig. 2) with a few isolated follicles in between the larger PP. All gradations of size occur and, at any given stage of development, new foci of PP development are added so that an exact count of PP is difficult since the PP grows in size continuously. Nevertheless, with every centimeter increase in CR length new visible foci of PP development appear, so that beginning with two incipient PP (Fig. 1) at ~15 wk gestation we counted 43 large and some small PP in the ~38 wk gestation fetal intestine.

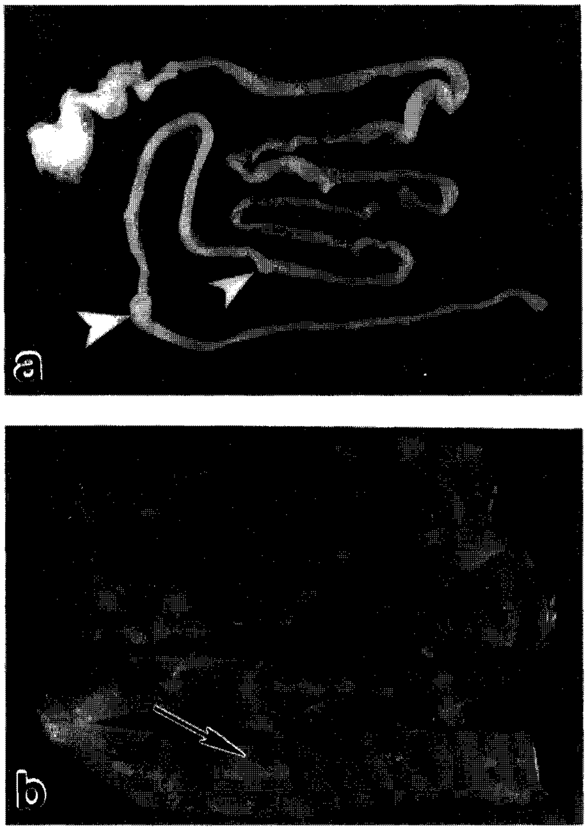

FIGURE 1 **a**. Alimentary canal of a human fetus (age ~15 wk) fixed in 3 % acetic acid within a few hours (?) of first appearance of gut associated lymphoid tissue. In two areas (white arrowheads) 2 – 3 lymph nodules were seen. The appendix has not yet developed. **b**. Part of the ileum of a human fetus (age ~34 wk) cut along the mesentery. Note the various sizes of Peyer's patches

### Hitsology

The structure of the lymphoid tissue of immature PP with few follicles differs from that of mature PP of older fetuses with many lymphoid follicles. The immature PP consists mostly of an extensive meshwork of reticulum fibers with developing lymphocytes that are scattered or occur in rows or in clusters (2 b, c, d). As the PP matures, the lymphocytes arrange themselves in the form of ovoid lymph nodules, only in one or two locations initially, and in several locations as the fetus grows. Occasionally, the lymph nodules of PP may occupy 90 % of the intestinal wall of mature PP of older fetuses.

### Immunocytochemistry

The 5$^{th}$ month of pregnancy seems to be earmarked for the commencement of development of surface markers of T and B lymphocytes (Fig. 2a – f) and of Natural Killer cells. The interesting finding here is that there is a graded series in which the surface markers of lymphocytes are expressed as the fetus grows from ~17 through ~22 wk gestation viz. absent → very faint positive in few cells in one or two locations → faint positive in a few cells of some areas of a follicle → distinctly positive in few cells in many areas → distinctly positive in numerous cells of many areas (Figs. 3).

Since the PP and the appendix were examined simultaneously, it was observed that not only does PP development precede that of the appendix, but the antigenic markers for T and B lymphocytes viz. CD19 B cells, CD9 pre- B cells, CD3 T lymphocytes, CD4 helper / inducer T cells, CD8 suppresser / cytotoxic T cells, and the CD 57 marker for Natural Killer cells, are expressed first in the Peyer's patches and thereafter in a later stage of development in the appendix. HLA-DR positive cells were seen in PP and appendix of all stages examined. While lymphocytes expressing HLA-DR are immature and sparse and not very clearly distinguishable (Fig. 2a) in the appendix of the 17.5 GW human fetus, they are numerous and distinct in the Peyers patches (Fig. 2b) of the same fetus It was observed that although the expression is dim, CD19 B cell marker is expressed, at ~17.5 wk whereas CD9 pre-B cell marker is expressed later, at ~20 wk gestation. We noticed that lymphocytes express CD3 and CD4 antigens at ~20 wk, while the antigens for the cytotoxic suppresser (CD8) and for Natural Killer cells (CD57) are the last to be expressed at ~22.5 wk. The range of antigenic expression in cells varied from dim to very high and also with the type of antigen. Cells expressing the CD3 antigen were present in clusters (Fig. 2e) while those expressing the CD 19 antigen were mostly scattered (2d). CD 9 positive pre B cells were present in rows (2c) and seldom, if at all, in small clusters in the immature PP of younger fetuses.

Another finding of consequence is that there is an ordered sequence in which the antigenic markers are expressed by lymphocytes viz. HLA-DR → CD19 → CD9 → CD3 → CD4 → CD8 → CD57. We observed that lymphocytes of immature PP express only B cells and the intestine in the PP region does not show distinction into all the layers found in the adult. Moreover, B cells appear to be distributed in rows in younger fetuses and in locations that show no similarity with adult lymphoid follicles. In older fetuses they occur in rows, or they may be scattered or may occcur in groups. Once the regulatory switch to expression of the repertoire of CD antigens is turned on at 22GW, CD3 lymphocytes far outnumber those expressing CD19 and CD9, and are more clustered and numerous in distribution in the appendix than in the PP (Figs. 2ef).

In older fetuses of ~22.5, ~23, ~30 and ~32 gestation week human fetuses, lymphocytes within PP and appendix were positive to all the antigenic markers tested. The CD9 and CD19 positive B cells may be scattered, or may occur in rows, or may be more concentrated in their distribution. HLA-DR positive cells were noticed both in the follicle associated epithelium as well as in the developing PP tissue in all stages examined. The most significant finding in the present studies is that the ~5 week fetal period from ~17.5 wk to ~22 wk represents a major growth phase in the development of surface markers of T and B lymphocytes in the mucosal immune system of the gut in humans.

### DISCUSSION

We report here the ontogeny of the gastrointestinal immune system with particular reference to the relationship of the Peyer's patches with the appendix in humans. Our findings are based on a large range, and also on closely graded stages of human development than those reported hitherto.

PP were first noticed at 15 wk gestation. Their development progresses at a rapid rate from two PP in the 15 wk fetus to 43 large and some small in the 38 wk fetus. They vary in size from lmm in younger fetuses to 5 – 6 cms in older fetuses. The only other record of counts of PP is of human fetuses, all more than 24 weeks gestation (Cornes, 1965a, b). The typical structure described for adult PP ( McGhee, 1993) consisting of follicle associated epithelium (FAE), dome, germinal centre of B cells, and a parafollicular area of T cells is not seen in human fetal PP. The reticular network structure of immature PP (Fig. Fig 2d) of younger fetuses is in sharp contrast with the compact mass of cells which form the follicles of mature PP of older fetuses. The spongy structure of immature PP probably serves as an efficient filtering device for the amniotic fluid along with the contained cellular debris that may be swallowed. Reynolds and Morris (1983) studied the evolution and involution of the PP

in fetal and postnatal sheep. They noted that around 8 wk gestation, the weight of PP was more than that of the thymus. They observed that one PP was 2.5 m long and that there were 1,000,00 PP follicles in lamb.

It is pertinent to mention here that several workers have stated that PP are not easily identifiable in the human intestine. "MacDonald *et al*, (1987) noted that the major difference between PP in humans and rodents and the principal factor hindering research in this area in humans is that whereas PP are grossly visible in rodent small bowel, they are not in adult human ileum". Orlic and Lev (1977) examined human fetal intestines and reported that they "did not find definitive lymphoid follicles in the wall of the small intestine through 20th week of gestation." They did not examine the intestine of older fetuses. As demonstrated here, this appears not to be the case. Ushio *et al.* (1985) used double contrast radiograph for fresh specimens and roentgenograms for fixed specimens and observed that Peyer's patches were discernible with great difficulty. Johnson (1989), stated with regard to the development of gut associated lymphoid tissue (GALT) that "although it has not been studied embryologically, GALT probably forms in much the same manner as other lymphoid organs". Due to the technique adopted in the present studies, not only were the PP identifiable without ambiguity but immunocytochemical studies could also be carried out. This is the first detailed report on the development of the immune system in humans that includes findings on the simultaneous appearance of surface markers of T and B cells in the Peyer's patches (including PP other than the terminal PP) as well as the appendix. We are the first to report that the expression of surface markers of T and B lymphocytes in the PP and the appendix commences at ~17.5 wk gestation and is completed by ~22 wk gestation and that this ~5 wk period represents a major growth phase for the gut in humans.

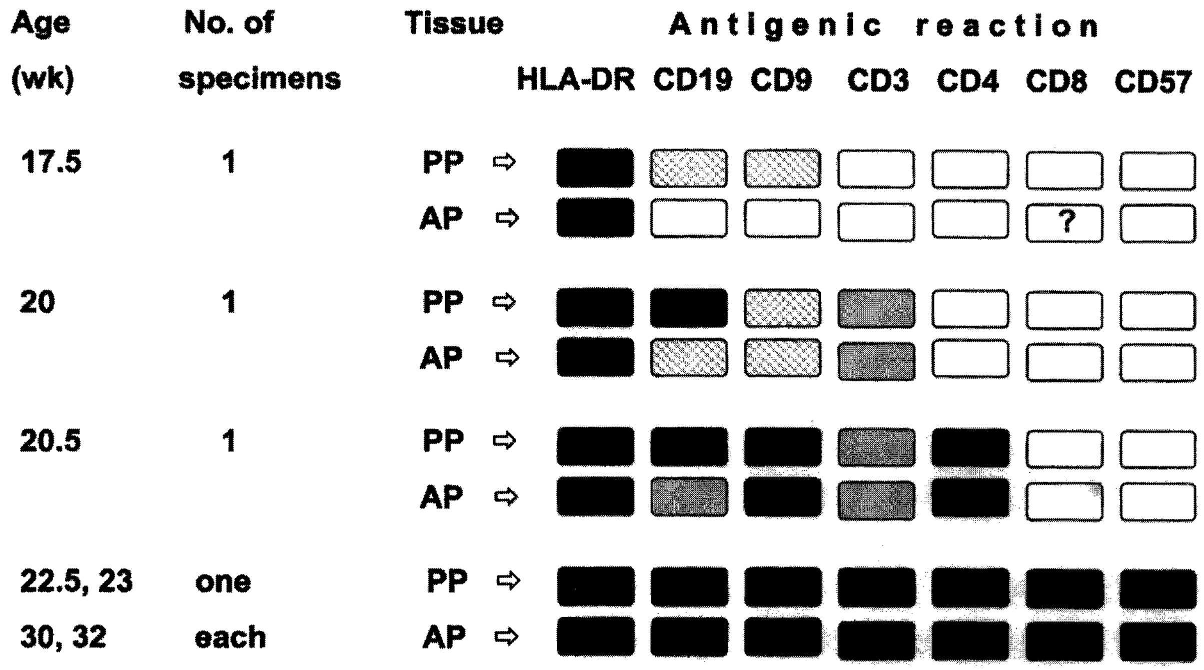

FIGURE 3 Stagewise expression of surface markers of lymphocytes. The B cell surface markers CD19 and CD9 are expressed first followed by the T cell markers CD3, CD4 (T helper), CD8 and the CD 57 marker for Natural Killer cells. During development the lymphocyte surface makers are expressed first in the PP and thereafter in the appendix. Empty box = no reaction; Empty box with? = doubtful reaction; cross – hatched box = very faint positive reaction in a few cells of some areas; dark gray box = positive reaction in many cells of some areas; black box = distinct positive reaction in many cells of larger areas

TABLE I Mouse monoclonal antibodies used as primary reagents

| *Designation* | *Specfcity* | *Isotype* | | *Working dilution* | *Source* |
|---|---|---|---|---|---|
| TAL-1B5 | HLA - DR | IgG1 | Purified Ig | 1:100 | Dako, A.S., Denmark |
| HNK - 1 | CD57 | IgGM | Purified Ig, | 1 : 100 | Sigma, U. S. A |
| UCHT1 | CD3 | IgG1 | Purified Ig, | 1 : 50 | Sigma, U. S. A. |
| MT310 | CD4 | IgG1 kappa | Purified Ig | 1 : 50 | Dako, A. S., Denmark |
| DK25 | CD8 | IgG1 kappa | Purified Ig | 1 : 50 | Dako, A. S., Denmark |
| DU - ALL1 | CD9 | IgG1 | Purified Ig | 1:50 | Dako A.S., Denmark |
| S J 25 C1 | CD19 | IgG1 | Purified Ig | 1 : 50 | Dako, A.S., Denmark |

Expression of a variety of genes in correct sequence and spatiotemporal pattern ensures normal development and histologic differentiation. Our findings demonstrate that HLA-DR is expressed by lymphocytes within PP and the appendix in all ontogenic stages examined. Little or no expression of CD antigens was noticed upto 20.5 wk gestation except dim expression of the CD19 antigen. The switch to higher intensity and heterogeneity of CD antigens expression occurs at 22 wk gestation. The regulatory signal that releases this switch and accelerates the expression of CD antigens on lymphocytes of PP and the appendix is not known. The pattern of developmental progression demonstrates that the sequence of expression of antigens is HLA-DR → CD19 → CD9 → CD3 → CD4 → CD8 → CD57 (Fig. 9). Besides, each of these antigens chronologically appears first in the lymphocytes of the PP and subsequently in those of the appendix.

Our findings, therefore, differ from those (Spencer *et al.*, 1986) where it has been reported that PP develop from a cluster of cells expressing CD4, very similar to those seen in adult tissue. However, we did notice very few (2 – 4) cells in one or two areas of the apendix of the 17.5 wk fetus, that were positive to the CD4 marker and are also of the opinion as that of Spencer *et al.* (1986) that they are not T cells but macrophages that are also CD4 positive. These disparaties must be viewed in the light of the fact that (1) the exact age of the fetus is difficult to ascertain (+/- 7 days) (2) there is a subtle gradation in the appearance of surface markers of T and B lymphocytes) (3) that frozen sections of only the terminal PP tissue and of very few specimens and also younger fetuses (n = 1, 19 wk; n = 4, 14 wk) were used by these workers.

We believe that the hypothesis that "complete maturation of the intestinal immune system seems to occur only after the intestine is challenged with both microbial and nutritional antigens" (Bandeira, et al, 1990) seems doubtful. Regulation and onset of expression of antigenic markers appears to be driven by internal triggers (Ferguson and Parrott, 1972, Diamond, 1986, Bandeira et al 1990, Mosley and Klein, 1992) since it is age dependant as is evident by data presented in the present findings.

In conclusion, it may be said that additional studies using a panel of antigenic markers of all stages of T and B cell development, simultaneously in the PP and appendix as well as other lymphoid organs, will throw considerable light on the intricacies of this hitherto little known aspect of ontogeny of the mucosal imune system in humans.

### MATERIAL AND METHODS

### Tissue specimens for morphology and histology

The intestines of 20 human fetuses, ranging in age from ~8 wk to ~33 wk gestation (~8, 10, 14, 15, 17, 18, 19, 20, 21, 24, 25, 29, 34, 38 wk gestation, of these 14, 15, 17, 19, 20 and 21 wk gestation – 2 specimens each, others one specimen each) were immersed in 3 % acetic acid for easy visualization of PP. Cryosections were cut for routine histology. Age in weeks was on the basis of crown rump length (CR

length). Ethical committee clearance was obtained and fetuses used were either from medical termination of pregnancies, or due to natural abortions or due to death after birth.

### Tissue for immunocytochemical staining

A novel technique was adopted to visualize PP other than the terminal PP for use in immunocytochemical staining. (Tissues of the last PP can be procured with ease since the latter lies in close proximity to the appendix on the anti mesenteric side and is easily identifiable, and has hence been the only tissue used for study during the past in most research publications). The intestine from 8 human fetuses ranging in age from ~17.5 to ~32 wk gestation was thoroughly cleaned of the meconium by passing cold Tris buffered saline through short pieces of intestine and by gentle pressure of hand applied from one end to the other. A 14-G needle was inserted into one end while an artery forceps closed the other end. Air was pumped into the intestinal piece so that it inflated like a balloon and a few PP were visible against a black background. A small part of the PP tissue was immersed in 3 % acetic acid for confirmation, while the other part was processed for cryosectioning.

### Immunocytochemisry

The appendix and PP tissue from 8 human fetuses (age ~17.5 wk to ~32 wk gestation) was cut and immersed in Polyvinylpyrrolidone (K – 30) and stored at 20° C. 4 – 6 μ sections were cut within 2 days, air dried at room temperature, fixed in chilled acetone for 10 minutes and stored at 20° C. For staining, sections were brought to room temperature, and subjected to endogenous peroxidase blocking for 30 minutes, followed by 20 minutes in 20 % goat serum to reduce background. Sections were treated with murine monoclonal antibodies for 1h (Table I). Secondary Ab used was goat anti mouse conjugated to biotin (Dako, A.S., Denmark). Incubation was for 1 h. The final incubation was in streptavidin peroxidase (Dako, A.S. Denmark), followed by 3, 3′ diaminobenzidine tetrahydrochloride (Sigma, U.S.A) as the chromogen. All immunostained sections were counter stained with hematoxylin. Tonsilar tissue was used as positive control while elimination of the antibody from sections served as negative control.

### *Acknowledgements*

We are grateful to the Council of Scientific and Industrial Research, Government of India, for their financial assistance [Project no. 37 (815) / 93 / EMR II].

### *References*

1. Ada, G..L. (1993) The induction of immunity at mucosal surfaces. Chapter 4. In Local Immunity in reproductive tract tissues. In: Local Immunity in Reproductive Tract Tissues. P.D. Griffin and A.M. Johnson., Ed. (Oxford University Press. WHO). pp. 73 – 76.
2. Asma, G.E.M., Vanden Bergh, R. L. and Vossen, J.M. (1983). Use of monoclonal antibodies in the study of the development of T lymphocytes in the human fetus. *Clin. exp. Immunol.* **53**: 429 – 436.
3. Baginsky A. (1882). Untersuchungen uber den Darmkanal des menschlichen Kindes. *Arch. Anat. Pathol.* **89**: 64 – 94.
4. Banchereau J., and Rousset F. (1992). Human B lymphocytes, phenotype, proliferation and differentiation. *Adv. Immunol.* **52** : 125 – 261.
5. Bandeira, A., Mota-Santos, T., Itohara, S., Degermann,S., Huesser,C., Tonegawa,S. and Coutinho, A.(1990). Localization of γ / δ Tcells to the intestinal epithelium is independent of normal microbial colonization. *J.Exp.Med.*, **172** : 239 –244.
6. Bjerke K., Brandtzaeg P., Fausa O. (1988). T cell association is different in follicle – associated epithelium of human Peyer's patches and villous epithelium. *Clin. exp. Immunol.* **74** : 270 – 275.
7. Bjerke K., Halstensen T. S., Johnson F., Pulford K., Brandtzaeg P. (1993). Distribution of macrophages and granulocytes expressing Lipoprotein (calprotectin) in human Peyer's patches compared with normal ileal lamina propria and mesenteric lymph nodes. *Gut.* **34** : 1357 – 1363.
8. Butzner, J.D. and Befus, A. D. (1989). Interactions among intraepithelial leucocytes and other epithelial cells in Intestinal Development and function. Chap. 37. In Human Gastro-intestinal Development. Lebenthal E., Ed. (New York: Raven Press), pp 749 – 775.
9. Cho D. (1931). Histological investigations of the digestive tract of the human fetus. II. Development of the small intestine. Jap. J. Obst. Gynec **14** : 324 – 330.
10. Cornes J.S. (1965). Number, size and distribution of m Peyer's patches in the human small intestine. Part I. The development of Peyer's patches. *Gut.* **6**: 225 – 229.
11. Cornes, J.S. *Ibid.* Part II. The effect of age on Peyer's patches. **6**: 230 – 233.
12. Diamond, J. M. (1986) Hard-wired local triggering of intestinal enzyme expression. *Nature*, **324** : 408.
13. Doe, W.F. (1989). The intestinal immune system. Gut. **30** (12) : 1679 – 1685.

14. Farstad I. N., Halstensen T. S., Fausa O., Brandtzaeg P. (1993). Do human Peyer's patches contribute to the intestinal v / o T-cell population? *Scand. J. Immunol.* **38**: 451 – 458.
15. Farstad I. N., Halstensen T. S., Fausa O., Brandtzaeg P. (1994) Heterogeneity of M – cell associated B and T cells in human Peyer's patches. *Immunology.* **83** : 457 – 464.
16. Farstad I. N., Halstensen T. S., Kvale D., Fausa O., Brandtzaeg P. (1995). Expression of VLA – 4 and L – selectin in human gut – associated lymphoid tissue (GALT). In Advances in Mucosal Immunology. Ed. Mestecky J. *et al.*, Ed (New York, Plenum Press) pp. 91 – 96.
17. Ferguson, A. and Parrot, D.M. (1972) Growth and Development of "antigen-free" grafts of fetal mouse intestine. *J. Pathol.*, **106** : 95 – 101.
18. Halstensen T.S., Scott H., Brandtzaeg P. (1989). Intraepithelial T cells of the TcR $\gamma$ / $\delta^+CD8^-$ and $V_\delta I$ / $J_\delta I^+$ phenotypes are increased in coeliac disease. *Scand. J. Immunol.* **30** : 665 – 672.
19. MacDonald T., Spencer J.., Viney J. L., Williams C. B., Walker-Smith J. A. (1987) Selective biopsy of human Peyer's patches during ileal endoscopy. *Gastroenterology.* **93** : 1353 – 1396.
20. Mahida Y.R, Patel S., Jewell D. P. (1989). Mononuclear phagocyte system of human Peyer's patches : an immunohistochemical study using monoclonal antibodies. *Clin. Exp. Immunol.* **75** : 82 – 86.
21. Mosley, R. L., Styre, D. and Klein, J.R. (1990) CD4+ CD8+ murine intestinal intraepithelial lymphocytes. *Int. Immunol.*, **2** : 361 – 365.
22. McGhee, J.R., Beagley, K.W., Fujihashi, K., Taguchi, T., Jiangchun Xu, Aicher, W.K., Mestecky, J. and Kiyono, H.. (1993). Chapter 2. Regulatory mechanisms in mucosal Immunity: roles for T – helper cell subsets and derived cytokines in the induction of IgA reponses. In: Local Immunity in Reproductive Tract Tissues. Ed. P.D. Griffin and A.M. Johnson.(Oxford University Press. WHO). pp 17 – 51.
23. Orlic D. and Lev R. (1977). An electron microscopic study of intraepithelial lymphocytes in human fetal small intestine. *Lab. Invest.* **37** (no.6) : 554 – 561.
24. Reynolds, J. D. and Morris, B. (1983). The evolution and involution of Peyer's patches in fetal post natal sheep. Eur. J. Imm. **13**: 627 – 635.
25. Spencer J., MacDonald T., Finn T., Isaacson G. (1986). The development of gut associated lymphoid tissue in the terminal ileum of fetal human intestine. *Clin. exp. Immunol.* **64** : 536 – 543.
26. Spencer J., Finn T., Isaacson P G. (1986). Human Peyer's patches : an immunohistochemical study. *Gut.* **27** : 405 – 410.
27. Ushio, K. (1985). Peyer's patches in jejunum and ileum excluding terminal section. A roentgenographic study using fresh and fixed surgical specimens. Stomach Intestine. 20 (7) : 747 – 758.
28. Yamamoto J. (1988). Lymphatic apparatus in the intestine : its macroscopic anatomy. *Stomach Intestine.* **23** : 1310 – 1314.

